# Identification of Epigenetically Modified Hub Genes and Altered Pathways Associated With Retinoblastoma

**DOI:** 10.3389/fcell.2022.743224

**Published:** 2022-03-10

**Authors:** Aditi Karmakar, Md. Maqsood Ahamad Khan, Nidhi Kumari, Nalini Devarajan, Senthil Kumar Ganesan

**Affiliations:** ^1^ Department of Structural Biology and Bioinformatics, CSIR-Indian Institute of Chemical Biology, Kolkata, India; ^2^ CSIR-IICB Translational Research Unit of Excellence (TRUE), Kolkata, India; ^3^ Centre of Bioinformatics, Institute of Interdisciplinary Studies, University of Allahabad, Prayagraj, India; ^4^ Academy of Scientific and Innovative Research (AcSIR), Ghaziabad, India; ^5^ Central Research Laboratory, Meenakshi Academy of Higher Education and Research, Chennai, India

**Keywords:** retinoblastoma, epigenetics, DNA methylation, miRNAs, differentially expressed genes (DEGs), hub genes, co-expression network analysis, biomarkers

## Abstract

Retinoblastoma (Rb) is the most common childhood malignancy initiated by biallelic mutation in *RB1* gene and driven by various epigenetic events including DNA methylation and microRNA dysregulation. Hence, understanding the key genes that are critically modulated by epigenetic modifications in *RB1* ^*−/−*^ cells is very important to identify prominent biomarkers and therapeutic targets of Rb. In this study, we for the first time have integrated various Rb microarray NCBI-GEO datasets including DNA Methylation (GSE57362), miRNA (GSE7072) and mRNA (GSE110811) to comprehensively investigate the epigenetic consequences of *RB* loss in retinoblastoma tumors and identify genes with the potential to serve as early diagnostic markers and therapeutic targets for Rb. Interestingly, the GEO2R and co-expression network analysis have identified three genes namely *E2F3, ESR1,* and *UNC5D* that are significantly deregulated by modified DNA methylation, mRNA and microRNA expression in Rb tumors. Due to their recognition in all epigenetic, transcriptomic, and miRNA datasets, we have termed these genes as “common genes”. The results of our integrative bioinformatics analysis were validated *in vitro* by studying the gene and protein expression of these common genes in Y79, WERI-Rb-1, Rb cell lines and non-tumorigenic retinal pigment epithelial cell line (hTERT-RPE). The expression of E2F3 and UNC5D were up-regulated and that of ESR1 was down-regulated in Rb tumor cells when compared to that in non-tumorigenic hTERT-RPE cells. More importantly, *UNC5D*, a potent tumor suppressor gene in most cancers is significantly up-regulated in Y79 and Weri Rb1 cells, which, in turn, questions its anti-cancer properties. Together, our study shows that *E2F3, ESR1,* and *UNC5D* may be crucially involved in Rb tumorigenesis and possess the potential to act as early diagnostic biomarkers and therapeutic targets of Rb.

## Introduction

Retinoblastoma (Rb), the most common childhood malignancy that develops in the retina of children under age five causes a great impact on the overall life of the affected child (Dimaras et al., 2015a). Rb pathogenesis can be hereditary or sporadic in nature. Hereditary Rb arises due to bilateral genetic mutations in *RB1* gene located in chromosome 13. On the other hand, sporadic Rb is non-heritable and arises due to unilateral somatic mutations in *RB1* gene of a single retinal cell after fertilization (Dimaras et al., 2015b). Rb globally affects 8,000 children per year with 69% cases in middle-income countries, 20% in low-income, and 11% in high-income countries (Dimaras et al., 2015a). Furthermore, the survival rate of these children is very low in developing countries owing to poor socioeconomic status. The major obstacle behind the high cure rate of Rb-affected children in these countries is the lack of awareness and the high cost for conventional Rb diagnostic procedures. Growing investigations on Rb affected children of developing countries state that the lack of diagnostic facilities at the genetic level in these countries is the prime cause for late diagnosis, vision loss, and treatment complications in Rb affected children (Chantada et al., 2010; Chintagumpala 2019; Chawla et al., 2021). Hence, the development of novel biomarkers and prominent therapeutic targets for the early diagnosis and precise treatment of Rb is of urgent priority.

It is well known that the Rb tumor development in the retina is initiated by the biallelic mutation in the retinoblastoma gene (*RB1*) leading to *RB1* ^*−/−*^, but this mutation alone does not result in retinoblastoma. Mounting studies show that cells lacking *RB1* gene undergo aberrant epigenetic alterations to become tumorigenic and metastatic (Benavente and Dyer 2015). In retinal cells, *RB1* gene loss promotes various epigenetic events including DNA methylation and microRNA regulation all of which ultimately culminate in the dysregulation of diverse oncogenic and tumor suppressor proteins that favor cancer pathogenesis (Greger et al., 1989; Reis et al., 2012; Singh et al., 2016). Indeed, hypermethylation of *RB1* promoter region due to the overlapping of CpG island 106 is found to be the major epigenetic mechanism underlying decreased retinoblastoma protein expression in Rb tumors (Choy et al., 2005; Livide et al., 2012) (Ohtani-Fujita et al., 1993). Furthermore, in the genome-wide promoter methylation analysis done on 19 primary Rb tumor tissues and six normal fetal retinae, 35 genes were found to be down-regulated due to promoter hypermethylation and 83 genes were found to be up-regulated due to promoter hypomethylation (Yazici et al., 2020). These pieces of evidence portray the significance of DNA methylation in the pathogenesis of Rb.

Apart from DNA methylation, microRNAs (miRNAs) have also been found to greatly influence the expression of various genes involved in retinoblastoma development (Golabchi et al., 2017; Lara Elis et al., 2019). miRNAs are the small non-coding RNAs (about 18–22 nucleotides long) that control the post-transcriptional expression of genes. By binding to the 3ʹ-untranslated regions of mRNAs, miRNAs promote their degradation and thus inhibit protein synthesis. Accumulating studies have identified various candidate miRNAs with high potential to regulate the key mRNA network in retinoblastoma (Mirakholi et al., 2013; Tong et al., 2019). Despite the identification of the major DNA methylation events and miRNAs that drive tumorigenesis in *RB1* ^*−/−*^ individuals, the key hub genes that are critically regulated by these epigenetic events remain obscure.

Hence, in the present study, we have systematically analyzed the multiple microarray datasets by employing potential integrative bioinformatics analysis and identified the key hub genes and molecular events involved in the onset and progression of retinoblastoma. Unlike routine integrative bioinformatics analysis that involves the combination of two major domains like gene expression profiles/epigenetic profile, genome profile/clinical profile, etc.(Yang et al., 2017; Zhang et al., 2019; Jin 2020), we for the first time have integrated three major domains of cancer biology including, DNA methylation, gene and microRNA expression to precisely predict the target proteins with the potential to serve as promising early diagnostic biomarkers of Rb. The results of the integrative bioinformatics analysis were further validated *in vitro* by performing comparative gene expression analysis between Y79, Weri Rb1 human Rb cell lines and non-tumorigenic retinal pigment epithelial cells (hTERT-RPE).

## Methods

### Raw Data Collection

Three sets of raw microarray data consisting of the expression profile of retinoblastoma were retrieved from Gene Expression Omnibus Database (GEO) (https://www.ncbi.nlm.nih.gov/geo/). These data consist of genome-scale DNA methylation profiling in Retinoblastoma by using Illumina Human Methylation 450 Bead Chip platform and samples from the normal human eye and five ocular diseases (GSE57362, 25 Samples) (Berdasco et al., 2017), Profiling of miRNAs in human retinoblastoma by using 2k custom array and RNA was extracted from two retinoblastoma and two matched normal retina samples (GSE7072, four Samples) (Huang et al., 2007), Distinct Gene Expression Profiles Define Anaplastic Grade in Retinoblastoma by using Affymetrix Human Gene 2ST Array and RNA was extracted from the 28 retinoblastoma and three matched normal retina samples (GSE110811, 31 Samples) (Hudson et al., 2018). Microarray datasets contain cancer and normal samples. The details of the datasets are given in Table 1.

**TABLE 1:** Summary of the microarray datasets and differentially expressed genes in each dataset.

Sr No	Cancer type	Accession No	Datasets	Sample	Upregulated genes	Downregulated genes	Total DEGs
1	Retino blastoma	GSE57362	DNA Methylation	Normal Retina = 10	100	167	267
				Retinoblastoma = 15			
2		GSE7072	miRNA	Normal Retina = 2	161	104	265
				Retinoblastoma = 2			
3		GSE110811	mRNA	Normal Retina = 3	33	737	770
				Retinoblastoma = 28			

**Selection of the significant DEGs, were made totally on the basis of P. value ≤ 0.05 and |log_2_FC|≥0.5 (in GSE57362) and |log_2_FC|≥2 (in GSE7072 and GSE110811).

### Data Processing

**FIGURE 1:**
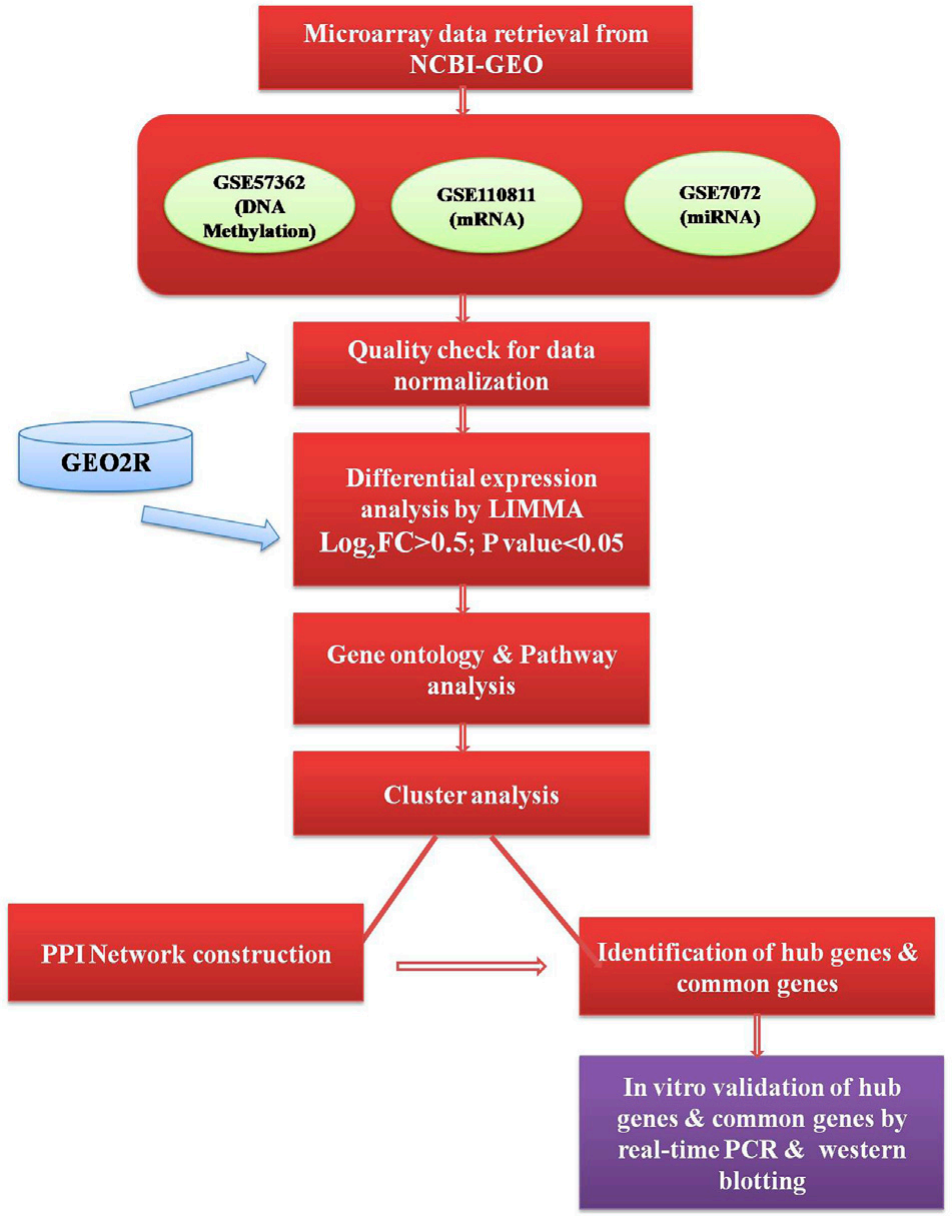
Outline of the workflow executed in an integrative analysis of multi-omics data.

GEO2R online software (https://www.ncbi.nlm.nih.gov/geo/geo2r/) was used to analyze the raw micro-array data for quality check and to identify the differentially expressed genes (DEGs). In GEO2R, the value distribution tab is used to assess the distributions of values across samples (median-centered) for the data normalization and each dataset was separately normalized by median normalization and the quality of the data accessed through box-plot. A brief outline of the workflow representing the datasets and methods used to conduct this study has been given in Figure 1.

### Identification of Differentially Expressed Genes

GEO2R is a web tool that allows differentiation among groups of samples that are differentially expressed across experimental conditions. The genes which are differentially expressed between Rb tumor samples and control samples were identified by the LIMMA package (Linear Models for Microarray data) using GEO2R. An adjusted *p*-value (p.adj)≤ 0.05 and logarithmic fold change (log_2_FC) ≥ 0.5 (GSE57362) and (log_2_FC) ≥ 2 (GSE7072 and GSE110811) were used as the threshold to identify DEGs. We have done an integrative analysis to identify the common DEGs among the three datasets were used in this study.

### Pathway Enrichment Analysis and Gene Ontology of DEGs

Gene ontology is used for an integrated research annotation and visualization to understand the cellular component, molecular function, and biological process concept of the specific genes. In this study, the PANTHER database (Protein Analysis Through Evolutionary Relationships) (http://www.pantherdb.org/tools/) was used to perform functional annotation and ShinyGO v0.61 (http://bioinformatics.sdstate.edu/go/) for KEGG (Kyoto Encyclopedia of Genes and Genomes) pathway enrichment analysis of differentially expressed genes.

### PPI Network Construction and Hub Genes Identification

The PPI co-expression networks of DEGs were created by using STRING (Search Tools For Retrieval of Interacting Genes/Proteins) database **(** https://string-db.org/). The database currently contains 24584628 proteins from 5,090 organisms. The network with a confidence score ≥0.7 was selected for PPI network construction, and the network was visualized and merged by using the Cytoscape (https://cytoscape.org/) software. In Cytoscape, each gene is represented as “node” and the strength of the co-expression relationship between genes is represented as “edge” and the number of interactions between the specific gene and other genes is represented as “degree”. Node with a degree >10 was selected as hub nodes. We used cytoHubba plugins in Cytoscape were used for identifying the hub genes and bottleneck genes.

### Cell Culture

The hTERT-immortalized retinal pigment epithelial cell line (hTERT-RPE- CRL-4000™), and WERI-Rb-1 retinoblastoma cell lines were purchased from ATCC. The Y79 retinoblastoma cell line (Riken: RCB1645 Y79) is a kind gift from Dr. Geeta K. Vemuganti, University of Hyderabad, Telangana, India. hTERT-RPE cells were cultured in ATCC-formulated DMEM: F12 medium supplemented with heat-inactivated 10% Fetal Bovine Serum (FBS) (Gibco™, Thermo Fisher Scientific) and 0.01 mg/ml Hygromycin B (Sigma-Aldrich Corp., St. Louis, MO, United States ). Y79 cells were cultured in Roswell Park Memorial Institute-1640 (RPMI-1640) (Gibco™, Thermo Fisher Scientific) medium supplemented with 10% Fetal Bovine Serum (FBS) (Gibco™, Thermo Fisher Scientific), l-Glutamine (Gibco™, Thermo Fisher Scientific) and antibiotics. Cell culture media was changed once every two days and cells were sub-cultured after reaching 80% confluence. All the experiments were carried out in triplicates by following good laboratory practices (GLP).

### Extraction of Total RNA and Quantitative Real-Time PCR

Total RNA was extracted from hTERT-RPE, Y79 and WERI-Rb-1 cells using RNAiso Plus reagent (TaKaRa, Japan) and quantification of RNA was carried out using Epoch 2 microplate reader, BioTek Instruments, United States . The extracted RNAs were then reverse transcribed into complementary DNAs (cDNA) using Prime-Script™ first strand cDNA Synthesis Kit (TaKaRa, Japan) according to the manufacturer’s protocol. Quantitative real-time PCR (qRT-PCR) was performed using TB Green™ Premix Ex Taq™ II (Tli RNaseH Plus) (TaKaRa, Japan). The relative mRNA level or fold change value for each gene in cancer cells compared to control was calculated using cycle threshold (Ct) value. The primers for gene expression analysis were designed using the NCBI Primer-BLAST tool and purchased from Integrated DNA Technology (Lowa, United States). The primer sequence details are given in Table 2.

**TABLE 2:** List of the oligonucleotide primers of the candidate genes used for the qPCR amplification.

Name of the candidate gene	Forward primer	Reverse primer
CDK1	5ʹ-CTT​GGC​TTC​AAA​GCT​GGC​TC-3ʹ	5ʹ-GGG​TAT​GGT​AGA​TCC​CGG​CT-3ʹ
CDK2	5ʹ-ATC​TTT​GCT​GAG​ATG​GTG​ACT​CG-3ʹ	5ʹ-TAA​AAT​CTT​GCC​GGG​CCC​AC-3ʹ
CCNB1	5ʹ-AAC​ATC​TGG​ATG​TGC​CCC​TG-3ʹ	5ʹ-CTG​ACT​GCT​TGC​TCT​TCC​TCA-3ʹ
RB1	5ʹ-TCC​CCG​GCG​CTC​CTC-3ʹ	5ʹ-TCA​AAC​TCA​AGC​CTG​ACG​AGA-3ʹ
CUL1	5ʹ-ACC​ACA​GAG​ATG​CGG​GTT​TG-3ʹ	5ʹ-AAA​GTC​GTC​CAG​TGC​AGC​AA-3ʹ
JUN	5ʹ-TGA​GTG​ACC​GCG​ACT​TTT​CA-3ʹ	5ʹ-TTT​CTC​TAA​GAG​CGC​ACG​CA-3ʹ
TP53	5ʹ-AAG​TCT​AGA​GCC​ACC​GTC​CA-3ʹ	5ʹ-CAG​TCT​GGC​TGC​CAA​TCC​A-3ʹ
HDAC1	5ʹ-CAT​CGC​TGT​GAA​TTG​GGC​TG-3ʹ	5ʹ-CCC​TCT​GGT​GAT​ACT​TTA​GCA​GT-3ʹ
MAPK1	5ʹ-ATT​TGT​CAG​GAC​AAG​GGC​TC-3ʹ	5ʹ-TCC​AAA​CGG​CTC​AAA​GGA​GT-3ʹ
PIK3CA	5ʹ-AGA​GCC​CCG​AGC​GTT​TC-3ʹ	5ʹ-TCA​CCT​GAT​GAT​GGT​CGT​GG-3ʹ
E2F3	5ʹ-CCA​AAA​ACT​CCA​AAA​TCT​CCC​TCA-3ʹ	5ʹ-GCA​CTT​CTG​CTG​CCT​TGT​TC-3ʹ
ESR1	5ʹ-TGG​GAA​TGA​TGA​AAG​GTG​GGA​T-3ʹ	5ʹ-GGT​TGG​CAG​CTC​TCA​TGT​CT-3ʹ
UNC5D	5ʹ-ATT​CGA​CTC​GGG​ACC​CTC​AT-3ʹ	5ʹ-ATT​GTC​AGT​TCC​TCG​GGC​AG-3ʹ
GAPDH	5ʹ-TCG​GAG​TCA​ACG​GAT​TTG​GT-3ʹ	5ʹ-TTC​CCG​TTC​TCA​GCC​TTG​AC-3ʹ

### Western Blot Analysis

Total protein was extracted from Y79, WERI-Rb-1and hTERT-RPE cells by using radio-immunoprecipitation (RIPA) lysis buffer (Himedia, India) supplemented with 1% phenylmethylsulfonyl fluoride (PMSF) (Roche, Germany) and 1% protease inhibitor cocktail (TaKaRa, Japan). Concentration of the respective proteins was measured by Bicinchoninic Acid (BCA) Protein Assay Kit (Thermo Fisher Scientific, United States ) using manufacturer’s protocol. Then proteins were separated by 10% SDS-PAGE gel, electrophoresed and transferred to nitrocellulose membrane with 0.45 µm pore size (Millipore, India). After being blocked with 5% non-fat milk (Himedia, India) for 1 h, the membranes were probed overnight with primary antibodies at 4°C. The following primary antibodies were used: anti-UNC5D/UNC5H4 antibody (1:1000 dilution, Cat.No. BS-11494R, Bioss), anti-E2F3 antibody (1:2000 dilution, Cat.No. BS-1722R, Bioss), anti-ESR1 antibody (1:2000 dilution, Cat.No. BS-2098R, Bioss) and anti-GAPDH antibody (1:4000 dilution, Cat.No.14C10, Cell Signaling Technology). Then the membranes were probed with Horseradish peroxidase (HRP) conjugated secondary anti-rabbit antibody (1:5,000 dilution, Cat. No. 114038001A, Genei, India) for 1 h at room temperature. Protein signaling was measured by enhanced chemiluminiscence (ECL) method and the images of the blots were quantified by using the ImageJ (Bio-Rad, United States) software.

### Statistical Analysis

All the data obtained from qRT-PCR & western blotting experiments were quantified for unpaired Student’s t-test using Graph pad prism 9.1.0 calculator and were represented in bar graphs using MS Excel. A value of p˂0.05 was considered as statistically significant.

## Results

### Identification of Differentially Expressed Genes (DEGs)

We identified DEGs between normal and tumor samples individually for each dataset by LIMMA package using GEO2R. Significantly differentially expressed genes were selected by qualifying the criteria P. value ≤ 0.05 and [log_2_FC]≥0.5 (GSE57362) and [log_2_FC]≥2 (GSE7072 and GSE110811). We henceforth found a total of 267 Differentially Methylated Genes (DMGs) in GSE57362, 265 Differentially Expressed miRNAs (DE-miRNAs) targets in GSE7072, and 770 DEGs in GSE110811.

**FIGURE 2:**
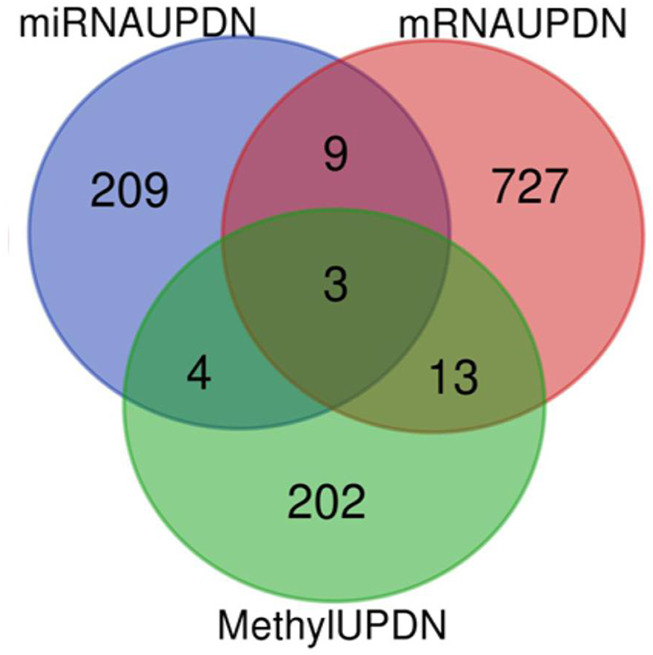
Venn diagram of the differentially expressed gens (DGEs) in the three datasets (DNA metylation, miRNA and mRNA).

The total numbers of upregulated and downregulated genes in each dataset were summarized in Table 1. The integrative analysis of all three datasets (DNA methylation, mRNA, and miRNA) showed that the three common genes (*E2F3, ESR1,* and *UNC5D*) were associated with Rb (Figure 2).

### Gene Ontology and Pathway Enrichment Analysis

**FIGURE 3:**
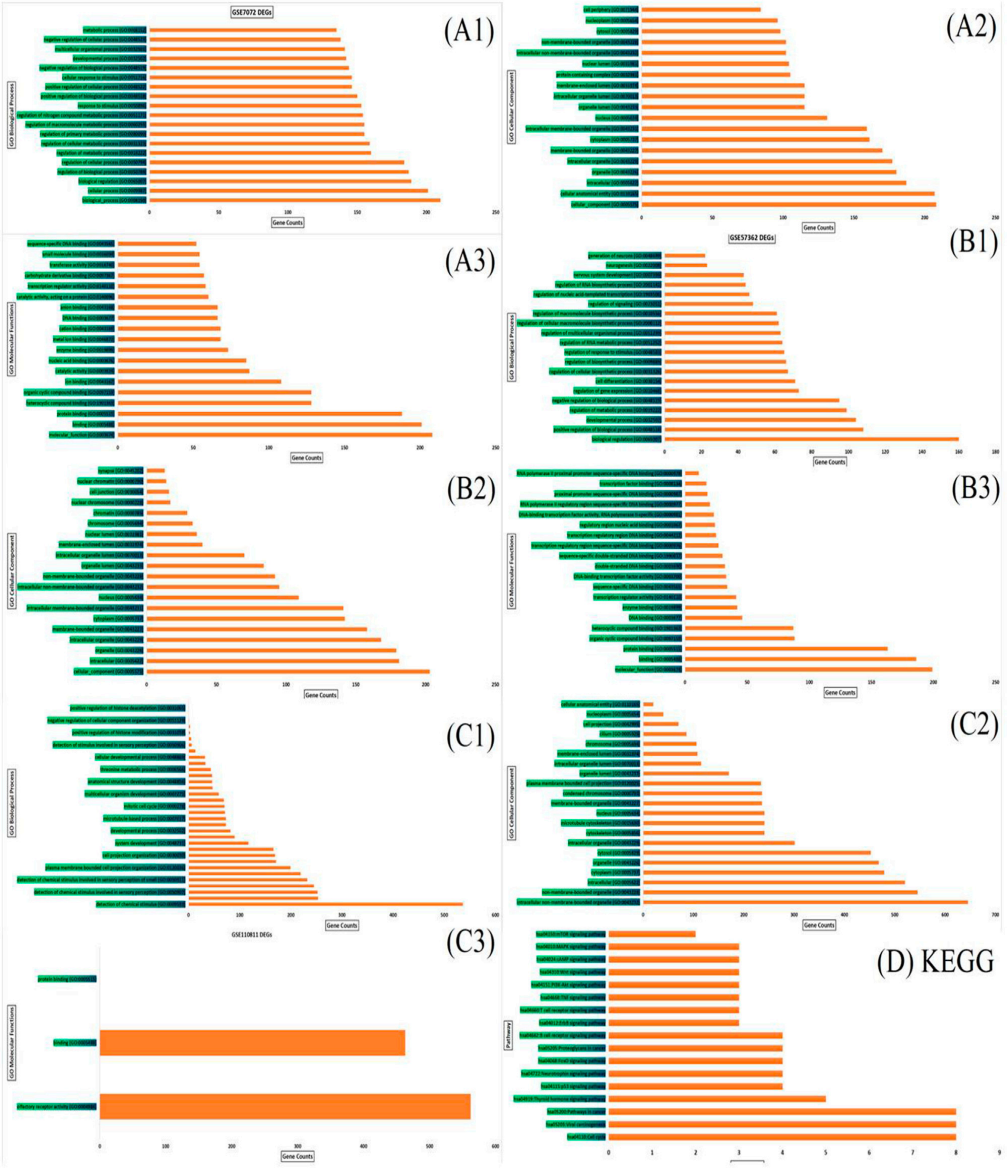
Functional enrichment analysis demonstrated enriched terms for the biological process, cellular component and molecular function of the three datasets **(A1–A3)** represent GO biological process, cellular components and molecular functions of GSE7072; **(B1–B3)** represent GO biological process, cellular components and molecular functions of GSE57362; **(C1–C3)** represent GO biological process, cellular components and molecular functions of GSE110811. **(D)** KEGG of top 17 significant pathway enrichment terms of hub genes in the three datasets.

The PANTHER and ShinyGO v0.61 were utilized to unwrap the biological activities and key regulatory pathways associated with the DEGs in DNA methylation (267 genes), miRNA (265 target genes) and mRNA (770 genes). Significantly enriched KEGG pathways and GO terms were identified based on P. value ≤ 0.05. The top 20 significant biological processes, molecular functions, and cellular components associated with the DEGs in the three datasets were shown in Figure 3A1–D . Table 3 shows hub genes involved in the top 17 significant molecular pathways in DNA methylation, miRNA, and mRNA datasets of Retinoblastoma. The results obtained showed that the p53 pathway, cell cycle, apoptosis and inflammatory pathways are the common pathways.

**TABLE 3:** Top 17 molecular pathways involved in retinoblastoma.

Serial No	Designation	Description	Count	p.value
1	hsa04110	Cell cycle	8	3.84E-10
2	hsa05203	Viral carcinogenesis	8	1.32E-08
3	hsa05200	Pathways in cancer	8	1.16E-06
4	hsa04919	Thyroid hormone signaling pathway	5	3.31E-05
5	hsa04115	p53 signaling pathway	4	1.82E-04
6	hsa04722	Neurotrophin signaling pathway	4	0.001015304
7	hsa04068	FoxO signaling pathway	4	0.001398004
8	hsa05205	Proteoglycans in cancer	4	0.004386821
9	hsa04662	B cell receptor signaling pathway	4	0.006133462
10	hsa04012	ErbB signaling pathway	3	0.009611516
11	hsa04660	T cell receptor signaling pathway	3	0.012558596
12	hsa04668	TNF signaling pathway	3	0.014290638
13	hsa04151	PI3K-Akt signaling pathway	3	0.019617316
14	hsa04310	Wnt signaling pathway	3	0.023116873
15	hsa04024	cAMP signaling pathway	3	0.045005064
16	hsa04010	MAPK signaling pathway	3	0.059756486
17	hsa04150	mTOR signaling pathway	2	0.056688768

### Co-expression Network Construction

**FIGURE 4:**
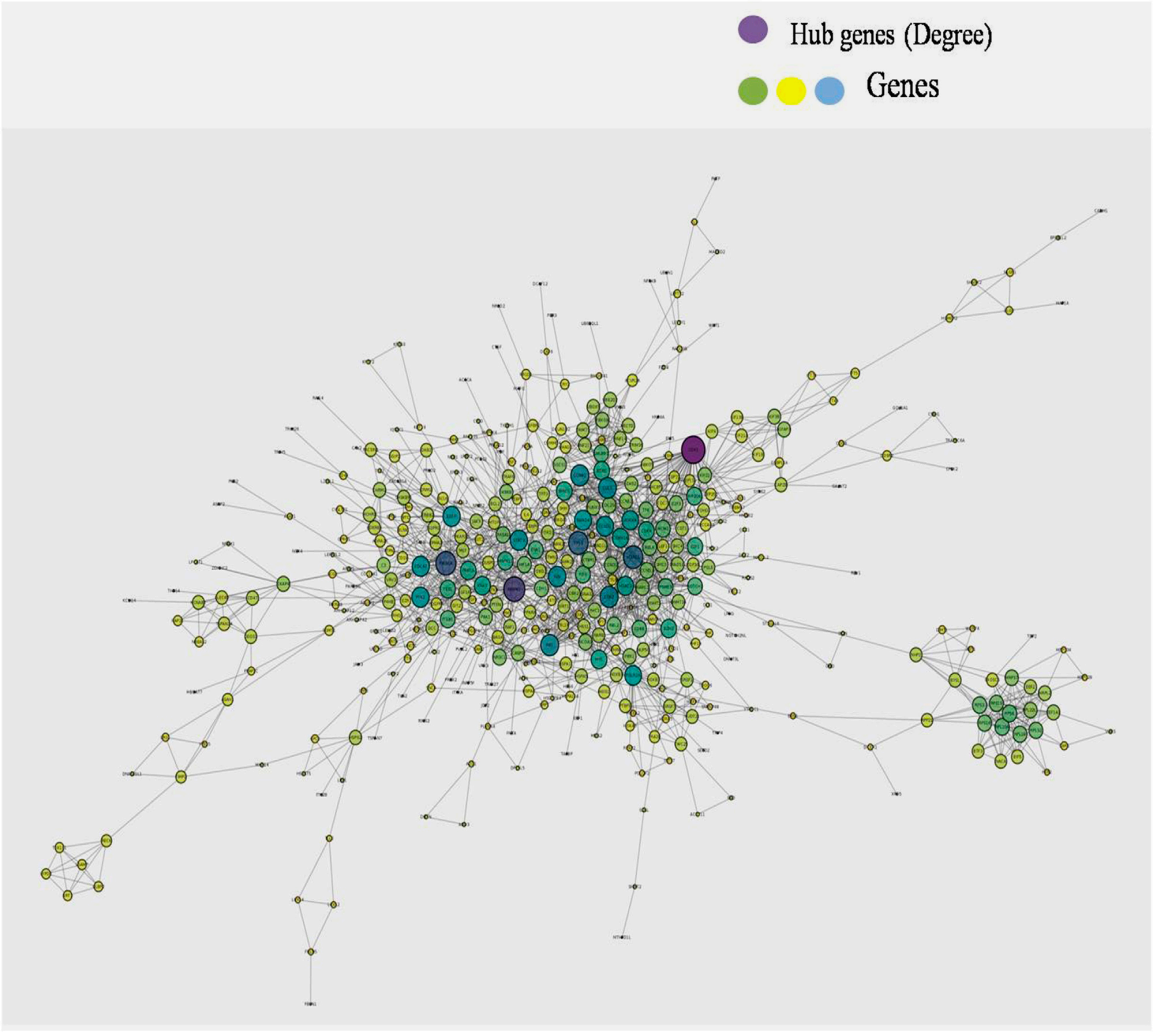
Combined co-expression network of DEGs in all the three datasets which has 498 nodes and 1642 interactions.

**FIGURE 5:**
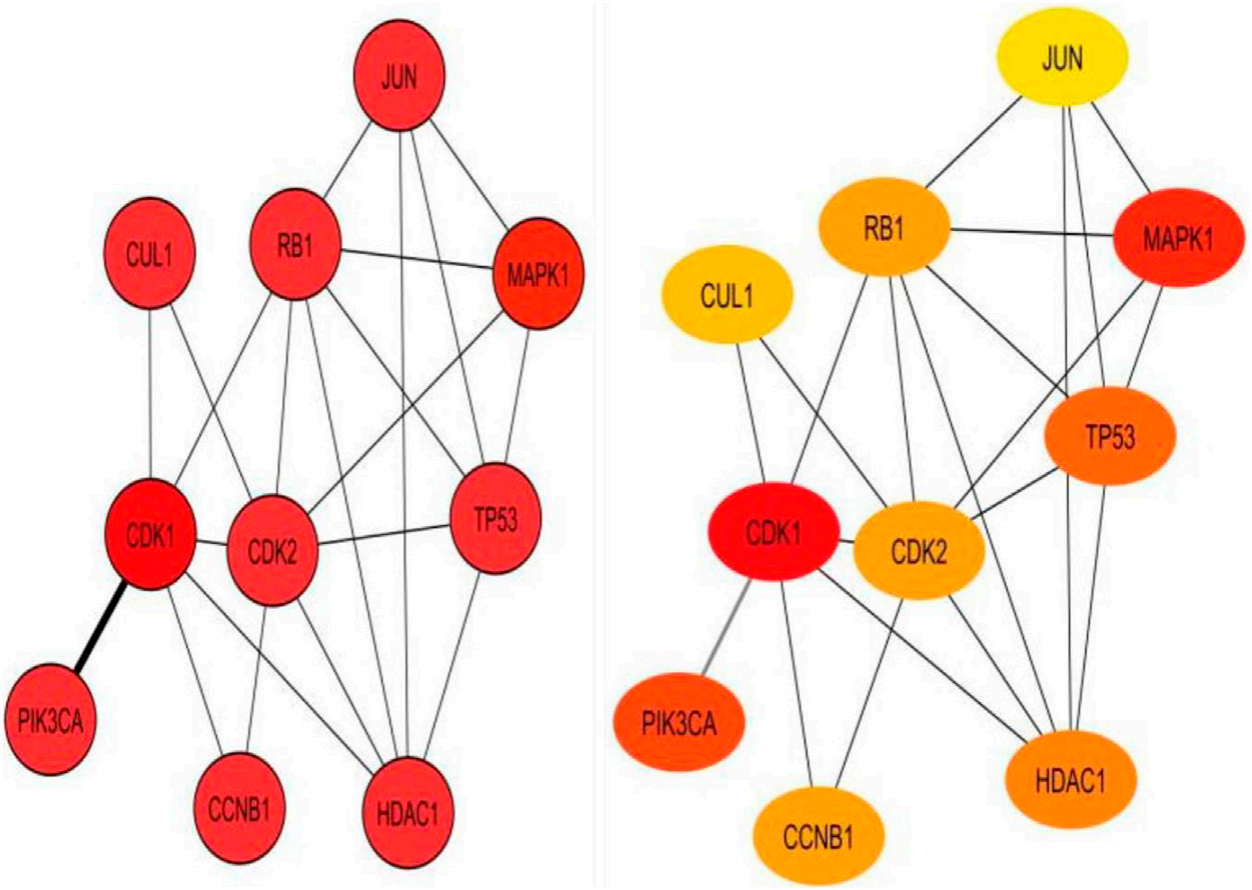
Top 10 hub genes identified from combined co-expression network of genes in the red module and degree basis module.

A single gene does not always contribute to disease progression by simply altering its’ expression level rather multiple genes interact with each other in a complex manner to cause the progression of any disease. Expression of a particular gene is the main criterion for the identification of DEGs and the interaction between multiple genes is not usually taken into consideration for routine DEGs analysis. The co-expression association between several genes is widely studied by constructing gene co-expression networks. We have found that the DNA methylation co-expression network has 63 nodes and 83 interactions, the miRNA co-expression network has 129 nodes and 424 interactions and the mRNA co-expression network has 243 nodes and 471 interactions as described in Table 4. A combined co-expression network of DEGs in all three datasets having 498 nodes and 1642 interactions were identified (Figure 4 **)**. Top 10 Hub genes, three Common and five Bottleneck genes identified from combined co-expression network analysis (Table 5). The hub genes in the red module and degree basis module were shown in Figure 5.

**TABLE 4:** Topological parameters used in co-expression network.

Dataset	Total DEGs	Nodes	Edges	Clustering-coefficient	Correlation	R^2^
GSE57362	267	63	83	0.771	0.840	0.781
GSE7072	265	129	424	0.691	0.966	0.761
GSE110811	770	243	471	0.789	0.975	0.872

**TABLE 5:** Hub genes, Common genes and Bottleneck genes found in co-expression network.

Type of genes	Name of genes	Degree
Hub Genes	CDK1	53
	MAPK1	44
	PIK3CA	40
	TP53	38
	HDAC1	37
	RB1	32
	CDK2	32
	CCNB1	32
	CUL1	31
	JUN	30
Common Genes	E2F3	40
	ESR1	42
	UNC5D	30
Bottleneck Genes	MAPK1	46
	PIK3CA	44
	PSME3	40
	NR3C1	32
	NHP2	27
	ITGB1	27

### Validation of Hub Genes and Common Genes

Quantitative real-time PCR was performed to check the differential expression of the hub genes and common genes in normal human retinal pigmented epithelial cells (hTERT-RPE) and Retinoblastoma (Y79 and WERI-Rb-1) cells. After analysis of the qPCR data, two hub genes i.e. CDK1 [*p* < 0.05 (Y79), *p* < 0.001 (Weri-Rb1)] and CCNB1 [*p* < 0.05 (Y79), *p* < 0.01 (Weri-Rb1)] and two common genes i.e. E2F3 (both *p* < 0.001) & UNC5D [*p* < 0.01 (Y79), *p* < 0.001 (Weri-Rb1)] were found to be significantly overexpressed in Rb cells whereas four hub genes i.e. RB1 (both *p* < 0.001), TP53 [*p* < 0.001 (Y79), *p* < 0.01 (Weri-Rb1)], JUN [*p* < 0.001 (Y79), *p* < 0.01 (Weri-Rb1)] & PIK3CA [*p* < 0.05 (Y79), *p* < 0.001(Weri-Rb1)] and one common gene i.e. ESR1 [*p* < 0.05 (Y79), *p* < 0.01 (Weri-Rb1)] were found to be significantly downregulated in Rb cells’ normalization. GAPDH was used as an endogenous control for normalization. The results of the qPCR experiment were shown inFigure 6A–D.

**FIGURE 6:**
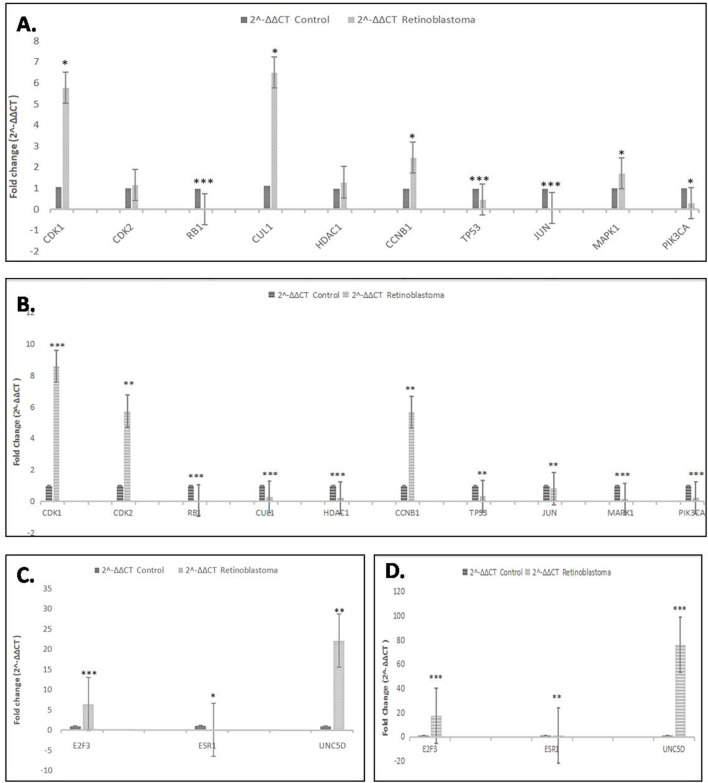
**(A,B)**: Real-time PCR analysis showing relative fold change of all 10 hub genes in Retinoblastoma cells (Y79 and WERI-Rb-1) as compared to control cells. GAPDH was used as endogenous control. **(C,D)**: Real-time PCR analysis showing relative fold change of all 3 common genes in retinoblastoma cells (Y79 and Weri-Rb1) as compared to control cells (hTERT-RPE). GAPDH was used as endogenous control. *p˂0.05; **p˂0.01 and ***p˂0.001.

**FIGURE 7:**
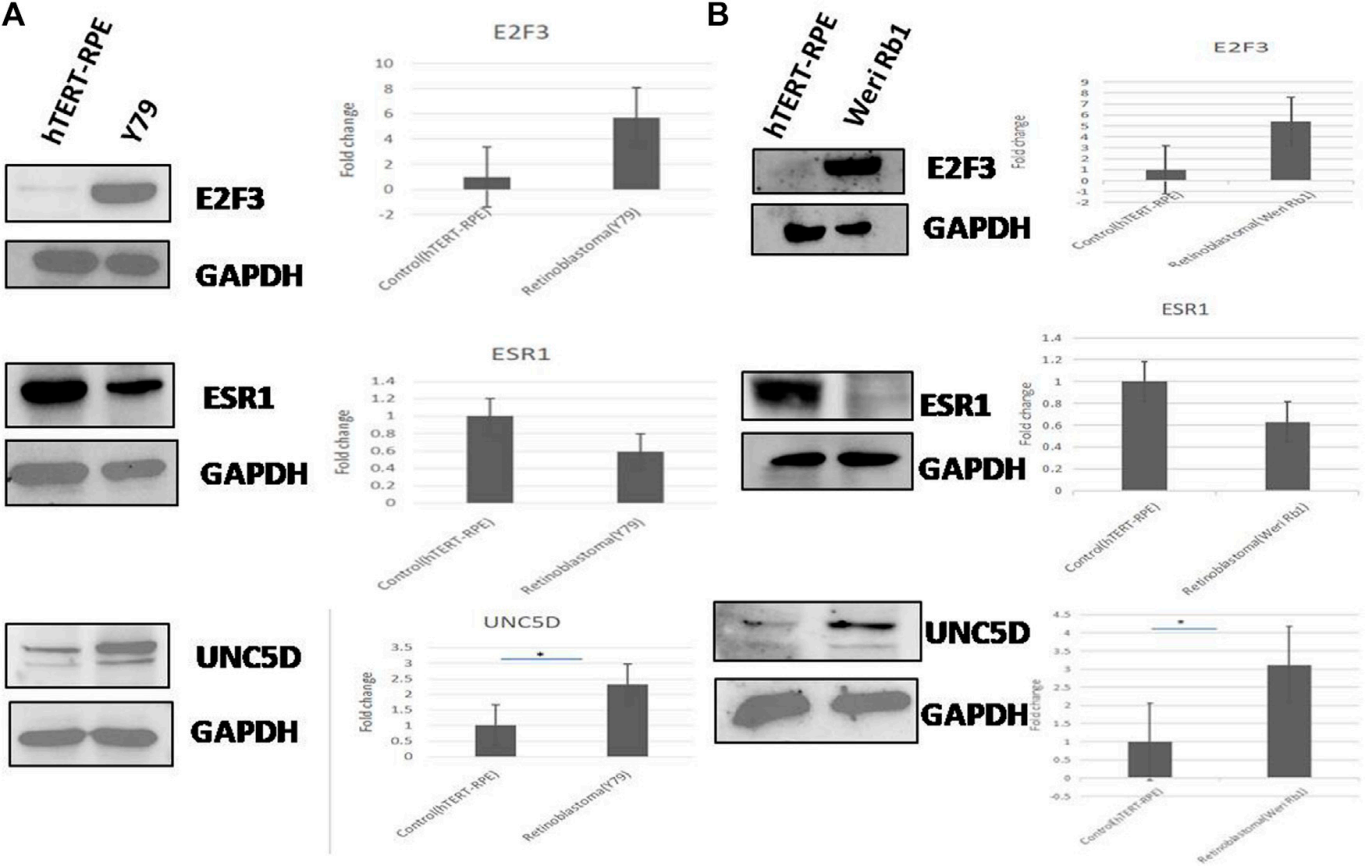
**(A,B)** The protein expression of all three common genes viz. E2F3, ESRI, and UNC5D was analyzed in two retinoblastoma cell lines (Y79 & Weri-Rb1) compared to normal retinal cell line (hTERT-RPE) by western blotting. Fold changes of the three proteins in retinoblastoma and normal cell lines obtained through densitometric analysis were represented in bar graphs. GAPDH was used here as loading control.

The protein expression of the common genes (UNC5D, E2F3 & ESR1) was checked in all 3 cell lines by western blotting. The expression of UNC5D protein was significantly upregulated and the expression of E2F3 was also increased in Y79 and WERI-Rb-1 retinoblastoma cell lines as compared to hTERT-RPE cells whereas the expression of ESR1 protein was downregulated in the above two retinoblastoma cell lines. Here, GAPDH was used as a loading control. The results of western blot experiment were shown in Figure 7A, B.

## Discussion

Integrative bioinformatics analysis is currently emerging as the most explicit approach to decipher the events of the tumor microenvironment and identify the key genes and signalling pathways underlying disease pathogenesis. Hence, this approach is widely used to predict the potent biomarkers and therapeutic targets of various cancers (Yang et al., 2017; Zhang et al., 2019; Jin 2020). In this study, we have integrated the expression profiles of three major domains of cancer biology including DNA methylation, mRNA, and miRNA in healthy and Rb cancer samples using individual microarray datasets available in NCBI-GEO to comprehensively understand the epigenetic consequences of Rb loss and map the key hub and common genes that play a crucial role in the development and progression of retinoblastoma tumors.

The GEO2R based LIMMA package analysis used to identify the DEGs between normal and Rb tumor samples has revealed 100 up-regulated and 167 down-regulated genes in the DNA methylation dataset, 161 up-regulated and 104 down-regulated genes in miRNA dataset, and 33 up-regulated and 737 down-regulated genes in mRNA dataset. Further, pathway enrichment analysis was performed using PANTHER and ShinyGO v0.61 to investigate the events modulated by these DEGs in Rb tumors. Our study results show that the pathways related to cell cycle progression (including p53 pathway), inflammation (including B cell receptor, ErbB, T cell receptor, Wnt, TNF, MAPK pathways), and cell proliferation (including PI3K/Akt/mTOR) are highly involved in Rb development. Finally, the three individual databases were merged by PPI network analysis using Cytoscape to identify the key hub genes regulated by all the three epigenetic events including DNA methylation, miRNA, and transcriptomics. Interestingly, our integrative bioinformatics analysis shows that 10 hub genes including *CDK1, CDK2, CCNB1, CUL1, RB1, TP53, HDAC1, JUN, PIK3CA,* and *MAPK1* are highly modulated by the epigenetic events in Rb tissues*.*

An important observation in our study is that three genes namely *E2F3, ESR1,* and *UNC5D* are identified among all the DNA methylation, miRNA, and mRNA datasets. Due to their recognition in all epigenetic, transcriptomic, and miRNA datasets, we have termed these genes as “common genes”. The identification of *E2F3, ESR1* and *UNC5D* in all the datasets analyzed clearly indicates their predominant role in Rb progression.

Most of the hub and common genes identified in Rb tissues are essentially involved in the cell cycle progression from G1 phase to S phase majorly via regulation by *RB1* gene. *RB1* is the tumor suppressor gene whose defective expression or biallelic mutation is central to the pathogenesis of Rb. *RB1* gene codes for retinoblastoma protein which exerts its anti-tumor effects by sequestering the activity of E2F transcription factors. In the active unphosphorylated state, retinoblastoma protein binds to the transcription factors of E2F family including E2 promoter binding protein dimerization partner (E2F-DP) and thereby inhibits their potential to activate genes underlying cell cycle progression. However, during the phosphorylated or mutated state, retinoblastoma protein is bereft of its potential to hold and inactivate E2F family transcription factors which in turn results in cell cycle progression from G1 to S phase in Rb cells (Dimaras et al., 2015b). Hence, in our bioinformatics analysis, it is well expected that *RB1* will emerge as the hub gene for Rb development and progression. However, the fascinating observation of our study is that the hub genes including *CDK1, CDK2, CCNB1,* and *HDAC1* and common genes including *E2F3* identified in Rb tissues are well-known oncogenes that trigger cell cycle progression in tumor cells in the absence of *RB1* gene (Cerqueira et al., 2007; Chenette 2010; Madhavan et al., 2009) (Ye et al., 2017) (Brehm et al., 1998). These observations suggest that the loss of *RB1* gene might trigger a series of epigenetic events that promote Rb tumor proliferation by inducing cell cycle progression. Hence, cell cycle progression could act as the key event that initiates and drives Rb pathogenesis.

Apart from cell cycle-associated genes, inflammatory pathways including Wnt, TNF, T cell receptor and B cell receptor are also found to be modulated in Rb tumors. These observations suggest an active role of inflammation in Rb tumors. It is well evident that inflammation augments the development and progression of various cancers including breast, lung, colon etc (Coussens and Werb 2010; Khandia and Munjal 2019; Greten and Grivennikov, 2020). However, on the contrary, mounting studies show that the activation of inflammatory pathways suppresses the growth of Rb tumors. In a recent study done by Sarver et al., 2021, early-onset Rb tumor cells have been found to express robust immune gene expression signature and accumulate diverse immune cells including monocyte, dendritic, T-lymphocyte, and macrophage in the tumor microenvironment. Fascinatingly, this immune cell infiltration inhibited the proliferation of Rb tumor cells thereby proving the immunoprotective role of inflammatory pathways against Rb tumor development (Sarver et al., 2021). In our study, the oncogenic inflammatory pathways including Wnt, TNF, T cell receptor identified by PANTHER and ShinyGO v0.61 analysis in Rb tumors are reported to induce apoptosis in Rb tumor cells (Cullinan and Brandt 2003; Hackam 2006; Mitra et al., 2012; Andersch et al., 2019). These evidences suggest that immunotherapy could emerge as a beneficial and effective strategy for Rb treatment.

The identification of *PIK3CA* and *MAPK1* genes as hub genes in Rb tumors suggest that cell proliferation is another hallmark event underlying Rb pathogenesis and progression. *PIK3CA* gene encodes a protein called as phosphatidylinositol-4,5-bisphosphate 3-kinase, catalytic subunit alpha (also known as p110α). This gene codes for PI3K, an enzyme that critically regulates cell proliferation through PI3K/Akt/mTOR pathway (Stelzer et al., 2016). In *Rb* ^*−/-*^ cells, *Rb* loss accompanied by the co-activation of PI3K/Akt pathway is found to be crucially involved in the initiation of Rb tumor development (Chakraborty et al., 2007; Cohen et al., 2009). Indeed, the immunohistochemical analysis of 27 human Rb tissue microarrays shows that p-Akt overexpression in highly proliferative tumors which in turn is indicative of its role in Rb progression (Xie et al., 2017). Hence, the identification of PI3K as hub gene in Rb tumors is evidential of the role of PI3K/Akt/mTOR signaling pathway in Rb tumor progression. *MAPK1* gene codes for MAPK1 enzyme which is also known as ERK2 and p42MAPK. Abnormal MAPK signaling in tumor cells activates series of events that eventually result in tumor cell proliferation, progression and ultimately metastasis (Guo et al., 2020). In Rb tumors, MAPK pathway has been found to be up-regulated by tripartite motif-containing protein 59 (TRIM59). TRIM59 is a potent oncogene whose overexpression is observed in a wide variety of human Rb cell lines including Y79, WERI-Rb-1and HXO-Rb44 indicative of its predominant role in Rb tumors. TRIM59 overexpression promotes the proliferation of Y79 human Rb cells and also attenuates their apoptosis both *in vitro* and *in vivo* majorly by increasing the phosphorylation of MAPK family of enzymes including pERK1/2, p-JNK1/2, p38 and also p-c-JUN (Wu et al., 2020). These evidences show the key role played by *MAPK1* gene in Rb progression.

The results of our integrative bioinformatics analysis were validated *in vitro* by evaluating the mRNA expression of hub genes in Y79, WERI-Rb-1Rb cell lines and non-tumorigenic hTERT-RPE retinal cell line. Our study showed a significant up-regulation in the expression of hub genes including *CDK1, CCNB1* & *CDK2* and dramatic down-regulation in the expression of hub genes including *RB1, TP53 and JUN* in Rb tumor cells. *CDK1, CCNB1* and *CDK2* are the key oncogenes that promote cell cycle progression in cancer cells (Cerqueira et al., 2007; Chenette 2010; Madhavan et al., 2009) (Ye et al., 2017) (Brehm et al., 1998). On the other hand, *RB1* and *TP53* are the tumor suppressor genes that inhibit cell cycle progression (Laurie et al., 2006; Hernández-monge et al., 2016). Taken together, the elevated expression of the hub genes that stimulate cell cycle progression and decreased expression of those that inhibit cell cycle progression in Y79 and WERI-Rb-1cells strongly suggest that cell cycle could be the major event underlying Rb tumor progression. Further importantly, the down-regulated expression of *JUN*, the inflammatory pathway activating gene (Schonthaler et al., 2011) in Y79 & WERI-Rb-1 cells suggests that inflammation plays a protective role against Rb tumor growth.

Further importantly, the gene and protein expression of common genes including *E2F3* and *UNC5D* were up-regulated and that of *ESR1* expression was down-regulated in Rb tumors when compared to that in non-tumorigenic hTERT-RPE cells. *E2F3* gene encodes the transcription factor E2F3 that is specifically bound by *RB1* in a cell-cycle-dependent manner. In the absence of *RB1*, *E2F3* acts as a key driver for cell cycle progression and hence, its overexpression is associated with tumor progression and poor prognosis in Rb patients (Cooper et al., 2006; Madhavan et al., 2009; Gao et al., 2017; Chen et al., 2019). Hence, our integrative bioinformatics and *in vitro* validation results suggest that the *RB1* gene loss in retinoblastoma patients could provoke epigenetic events which in turn dysregulate the expression of E2F3 and thus promote Rb development in retinal cells.

*ESR1* gene codes for estrogen receptor alpha (ERα), a nuclear receptor agonist for estrogen hormone (Carrillo-moreno et al., 2019). In recent years, ESR1 therapy garners more attention in the effective treatment of breast cancer (Dustin et al., 2020). A study done by Caliguiri and colleagues show that pRb loss in tumor cells attenuates the expression of ESR1 protein by the activation of the proteasome pathway (Caligiuri et al., 2013). In our study as well, the gene and protein expression of *ESR1* in Y79 and WERI-Rb-1tumor cell lines are down-regulated which in turn may be due to the loss of *RB* tumor suppressor gene. Furthermore, this is the first study that has revealed the expression of ESR1 in Rb cells. Hence, detailed analysis on the role of ESR1 in Rb tumors is highly warranted to unravel its role in Rb pathogenesis.

*UNC5D* gene codes for the tumor suppressor protein *UNC5D* (also known as *UNC5H4*) which acts as netrin NTN4 receptor in neuronal cells. It plays a major role in cell-cell adhesion and cell migration. Also, *UNC5D* serves as a dependence receptor needed for triggering apoptosis in DNA-damaged cells when it is not bound by netrin ligand. Its expression is reported to be significantly attenuated in various cancers (Lu et al., 2013; Wang et al., 2014). However, in this study, *UNC5D* gene expression is found to be remarkably high in Y79 and WERI-Rb-1retinoblastoma cells when compared to that in hTERT-RPE cells and other cancer cell lines including HeLa (human cervical cancer cell line), HCT116 (human colon cancer cell line) and SH-SY5Y (human neuroblastoma cell line) (data not shown). This specific up-regulation of *UNC5D* genes in Rb cancer cells is very interesting and also intriguing. Our study results suggest that *UNC5D* might act in a duplicitous manner in different cancer types. While acting as a tumor suppressor gene in most cancers, *UNC5D* might behave like an oncogene in Rb tumors. This unique overexpression of *UNC5D* gene in Rb tumor cells suggests that *UNC5D* might act as a novel and promising biomarker for the early diagnosis of retinoblastoma.

Another most interesting observation in our study is that a few of the hub genes of Rb tumors including *CDK1, CDK2, CCNB1, HDAC1* and *UNC5D* are reported to play a key role in the pathogenesis of osteosarcoma, the most common secondary cancer in Rb patients. It is proven that the hereditary Rb survivors are at high risk for developing osteosarcoma due to shared genetic alterations (Hansen et al., 1985; Kleinerman et al., 2012; Fujiwara et al., 2015). The association of the hub genes identified in Rb tumors with the pathogenesis of osteosarcoma sheds light on the mechanism underlying shared epigenetic modifications between retinoblastoma and osteosarcoma tumors in hereditary Rb survivors. Deciphering the role of these hub genes in the tumorigenesis of osteosarcoma in *RB1* ^*−/−*^ osteoblast cells will aid in developing prominent biomarkers for the regular monitoring of the hereditary Rb survivors for osteosarcoma development.

To summarize, the integrative bioinformatics analysis has identified ten hub genes including *CDK1, CDK2, CCNB1, CUL1, RB1, TP53, HDAC1, JUN, PIK3CA,* and *MAPK1* and three common genes including *E2F3, ESR1* and *UNC5D* in Rb tumors. Among them, the mutation of *RB1* gene is the prime cause for the pathogenesis of hereditary and sporadic retinoblastoma (Dimaras et al., 2015a). It is well known that cell cycle activation is the key event underlying the *RB1* deficiency in retinal cells through the inactivation of p53 pathway along with the activation of E2F3, CDKs and HDAC1 in children with both hereditary and sporadic Rb [Dimaras et al., 2015b]. However, the identification of *JUN, PIK3CA, MAPK1* and *UNC5D* as hub genes in our study has thrown light on the role of inflammation, cell adhesion and cell proliferation in Rb tumors. More importantly, our investigation on the expression of hub and common genes in Y79, WERI-Rb-1and hTERT-RPE cells has validated the results of our integrative bioinformatics analysis. While the expression of cell cycle inducing genes including *CDK1, CDK2* and *E2F3* are significantly up-regulated, cell cycle inhibitory genes including *RB1, TP53* are significantly down-regulated in Rb cancer cells cultured *in vitro*. Further importantly, *UNC5D*, a potent tumor suppressor gene whose protein expression is down-regulated in various cancer cells including HeLa, HCT116 and HuH cancer cells is found to be significantly up-regulated in Y79 and WERI-Rb-1Rb tumor cells. These findings question the protective role of *UNC5D* in cancer development and suggest that *UNC5D* might act in a duplicitous manner in different cancers. The results of the present study warrant detailed analysis using animal models and clinical samples to decipher the molecular mechanisms underlying Rb pathogenesis, elucidate the role of *UNC5D* in Rb tumors and identify if *E2F3, ESR1* and *UNC5D* could act as novel and promising targets for early diagnosis and treatment of Rb.

## Data Availability

The datasets presented in this study can be found in online repositories. The names of the repository/repositories and accession number(s) can be found in the article/supplementary material.
